# Study on bioactive compounds of microalgae as antioxidants in a bibliometric analysis and visualization perspective

**DOI:** 10.3389/fpls.2023.1144326

**Published:** 2023-03-28

**Authors:** Ning Yang, Qingyang Zhang, Jingyun Chen, Shilin Wu, Ran Chen, Liping Yao, Bailei Li, Xiaojun Liu, Rongqing Zhang, Zhen Zhang

**Affiliations:** ^1^ Zhejiang Provincial Key Laboratory of Applied Enzymology, Yangtze Delta Region Institute of Tsinghua University, Jiaxing, China; ^2^ College of Fisheries and Life Science, Shanghai Ocean University, Shanghai, China; ^3^ Qingyuan County Edible Fungus Industry Center, Lishui, China

**Keywords:** microalgae, antioxidants, bioactive substances, bibliometrics, citespace

## Abstract

Natural antioxidants are more attractive than synthetic chemical oxidants because of their non-toxic and non-harmful properties. Microalgal bioactive components such as carotenoids, polysaccharides, and phenolic compounds are gaining popularity as very effective and long-lasting natural antioxidants. Few articles currently exist that analyze microalgae from a bibliometric and visualization point of view. This study used a bibliometric method based on the Web of Science Core Collection database to analyze antioxidant research on bioactive compounds in microalgae from 1996 to 2022. According to cluster analysis, the most studied areas are the effectiveness, the antioxidant mechanism, and use of bioactive substances in microalgae, such as carotene, astaxanthin, and tocopherols, in the fields of food, cosmetics, and medicine. Using keyword co-occurrence and keyword mutation analysis, future trends are predicted to improve extraction rates and stability by altering the environment of microalgae cultures or mixing extracts with chemicals such as nanoparticles for commercial and industrial applications. These findings can help researchers identify trends and resources to build impactful investigations and expand scientific frontiers.

## 1 Introduction

Antioxidants are biological macromolecules that shield organisms or physical products from oxidative radicals (de Souza et al., 2019). The significance of antioxidants in reducing oxidative processes and the harmful effects of reactive oxygen species (ROS) is crucial in both food systems and the human body (Cakmakci et al., 2015). Since the 1970s, tert-butyl hydroquinone (TBHQ), butylated hydroxytoluene (BHT), EDTA, and other compounds have become some of the most widely utilized chemical antioxidants in the food business (Ngo et al., 2012), but this could cause liver damage and even cancer (Thannickal and Fanburg, 2000; Waris and Ahsan, 2006). Nowadays, the pursuit of health has made it a global trend to look for natural antioxidants that can replace chemical antioxidants and are non-toxic, noncarcinogenic, and biodegradable. In this context, microalgae present a promising alternative to meet the rising demand for sources of natural antioxidants. According to the worldwide algae market analysis report 2019–2025, the market for algae is predicted to increase by US$414.8 Thousand, with a compound growth rate of 6.6% (Uma et al., 2022).

Microalgae play a fundamental role in ecosystems (Guedes et al., 2011). Microalgal biomass and intracellular compounds have been studied and used as renewable natural resources for a wide spectrum of applications. However, microalgal biomass is typically utilized to produce biofuels or create powders and pills to strengthen immunity and prevent disease (Chacon-Lee and Gonzalez-Marino, 2010), making it challenging to include them directly in bulk meals (Kova et al., 2013). This is due to their color and odor. Additionally, studies on microalgal biomass have only included well-known species like *Spirulina, Chlorella and Dunaliella* (Gouveia et al., 2008). When exposed to light, carbon dioxide, and other external stressors, the abundance and variety of bioproducts they can produce, including high-quality proteins, long-chain polyunsaturated fatty acids, carotenoids, vitamins, minerals, and phenolics (Plaza et al., 2009). Microalgae-derived bioactive compounds have been investigated for their varied activities. Chronakis (Chronakis, 2001) researched *Spirulina* obturation proteins in 2001 and determined that microalgae proteins have superior gelling capabilities. Ursu et al. (Ursu et al., 2014) studied *Chlorella* proteins’ ability to emulsify in 2014. According to several studies, lutein and zeaxanthin are the significant pigments that cause the human macula to turn yellow and maintain proper visual function (Le and Xiao-Ming, 2010). In addition, various microalgal bioactive substances have specific antioxidant properties and may be a reliable source of natural antioxidants (Li et al., 2007). Such bioactive molecules serve as important foundations for biotechnological applications, particularly in the pharmaceutical, nutraceutical, and cosmetics sectors (Rani et al., 2021). For instance, Ayna (Ayna, 2020) researched the *in vitro* anticancer effects of beta-tocopherol, alpha-carotene, and ascorbic acid on PC-3 prostate cancer cells. These compounds play the role of oxidants in prostate cancer by reducing cell viability and increasing ROS production and lipid peroxidation (LPO). Carotenoids can be used as a natural antioxidant in anti-aging skin care products in the cosmetics industry, in addition to being incorporated as a natural pigment to color cosmetics (Foo et al., 2021).

The field of study on microalgal antioxidants is flourishing, and each year there are more research articles in this area. However, it is a challenge for researchers to locate research gaps and hotspots among the numerous relevant articles. Bibliometrics, which Alan Pritchard established in 1969 and uses mathematical and statistical methods to quantitatively analyze all sorts of knowledge vectors, is considered as a potent instrument to handle this challenge with the emergence of current information technology and statistics (Merigo et al., 2016). The most widely used bibliometric tools in use today are VOSviewer, Gephi, CiteSpace, and Bibexcel. Numerous publications about microalgae and bioproducts antioxidants have recently been published, including the following: management of oxidative stress by microalgae (Cirulis et al., 2013); microalgae under environmental stress as a source of antioxidants (Gauthier et al., 2020); astaxanthin in skin health, repair, and disease (Davinelli et al., 2018); microalgae integrated into innovative food with potential health benefits (Caporgno and Mathys, 2018). There aren’t many bibliometric studies on microalgae, nevertheless, and there haven’ t been any studies that illustrate the antioxidant qualities of the bioactive components of microalgae. Through the use of other visual analysis tools like CiteSpace and VOSviewer, this paper offers a quantitative and visual analysis of the literature about antioxidant research on highly valuable microalgal metabolites, as well as a detailed discussion of a number of hotspots and significant research topics to comprehend the knowledge structure, research hotspots, trends, and research frontiers (Wang et al., 2022).

## 2 Data and methods

### 2.1 Data acquisition

Reliable sources must be retrieved in order to guarantee the accuracy of the knowledge mapping (Xia and Duan, 2022). The Web of Science (WOS) database was used as the data source in this article. The WOS database search selected “Web of Science Core Collection” and input in the search formula: “(TS=(Antioxida*) OR TS=(Anti-oxida*)) AND TS=(Microalgae)”. Any terms with the same root as the word are guaranteed to appear in the search result when a word is finished with an asterisk, thus, this strategy searched for papers that contain the word antioxidant/antioxidant and its derivatives in their title, abstract, or keywords. According to our search formula, we found that Hirayama, S. et al. (Hirayama et al., 1996) released the first publication on microalgae antioxidant activities study in 1996, which can be retrieved from WoSCC. Consequently, the search data’ s time frame was set from January 1996 to November 2022 (the search date was 24 November 2022). The final result was the extraction of 2093 publication records, each of which had the following information: author, title, source document, abstract, and cited references.

### 2.2 Bibliometric methods for collaboration and citation networks

All visual analyses in this study were performed using VOSviewer 1.6.18.0, CiteSpace 6.1.R3, and Gephi, respectively. VOSviewer is a free JAVA-based software developed in 2009 by VanEck and Waltman from the Centre for Science and Technology Studies (CWTS) at Leiden University in the Netherlands (van Eck and Waltman, 2010), and this paper primarily uses VOSviewer for keyword co-occurrence and density analysis. CiteSpace is a multifaceted, dynamic visual analysis software developed by Chen CM (Chen, 2004) in 2004, focusing on the analysis of the underlying knowledge contained in the scientific literature (Sun et al., 2019). CiteSpace is used in this work for subject, country/region, institution, co-author, highly cited literature, keyword clustering, and burstness analysis. The analysis of hotspots in this research area is made possible by the keyword co-occurrence and clustering methods. This analysis clarifies how the research on antioxidants in bioactive substances in microalgae has developed, gives a proper understanding of the research spectrum, and serves as a guide for the field’s future development.

### 2.3 Indicators and algorithms

CiteSpace is used to analyze co-occurrence or collaborative networks from 1996 to 2022, with a one-year time slice. The selection criteria were each article’s top 50 cited or widely used sections (Tao et al., 2022). CiteSpace employs the Cosine algorithm to determine the strength of the relationships between keywords during the clustering process and the log-likelihood ratio (LLR) method to name each cluster. The cosine algorithm (Equation 1) groups keywords based on similarity. And the latter (Equation 2) proposes nomenclature to identify hotspots for current research in a given field.


(1) $$Cosine\left(c_{ij},\;f_{i},\;f_{j}\right)=\frac{c_{ij}}{\sqrt{f_{i}\times f_{j}}}$$

In Equation 1, *i* and *j* represent two subject terms, respectively, *c_ij_* is the number of co-occurrences of *i* and *j*; *f_i_* denotes the number of occurrences of *i*; *f_j_* denotes the number of occurrences of *j*.


(2) $$LLR=log\left(\frac{p\left(C_{i}/V_{ij}\right)}{p\left({\bar{C}}_{i}/V_{ij}\right)}\right)$$

LLR is the log-likelihood ratio of *w_i_* for category *C_j_*; *p*(*C_i_*/*V_ij_* and $p\left({\bar{C}}_{i}/V_{ij}\right)$ are the density functions in categories *C_j_* and ${\bar{C}}_{i}$ , respectively.

## 3 Results and discussion

The findings of this research are detailed in four sections that make up this section. Regarding annual publication volume, appropriate fields, countries, and institutions, Sections 3.1 and 3.2 give an overview of the current research and collaboration on antioxidants in microalgae and their byproducts. The highly cited papers are listed in Section 3.3, which also conducts a cluster analysis using the highly cited articles’ keywords. The research frontiers are analyzed using keywords in section 3.4.

### 3.1 Literature published analysis

**Figure 1:**
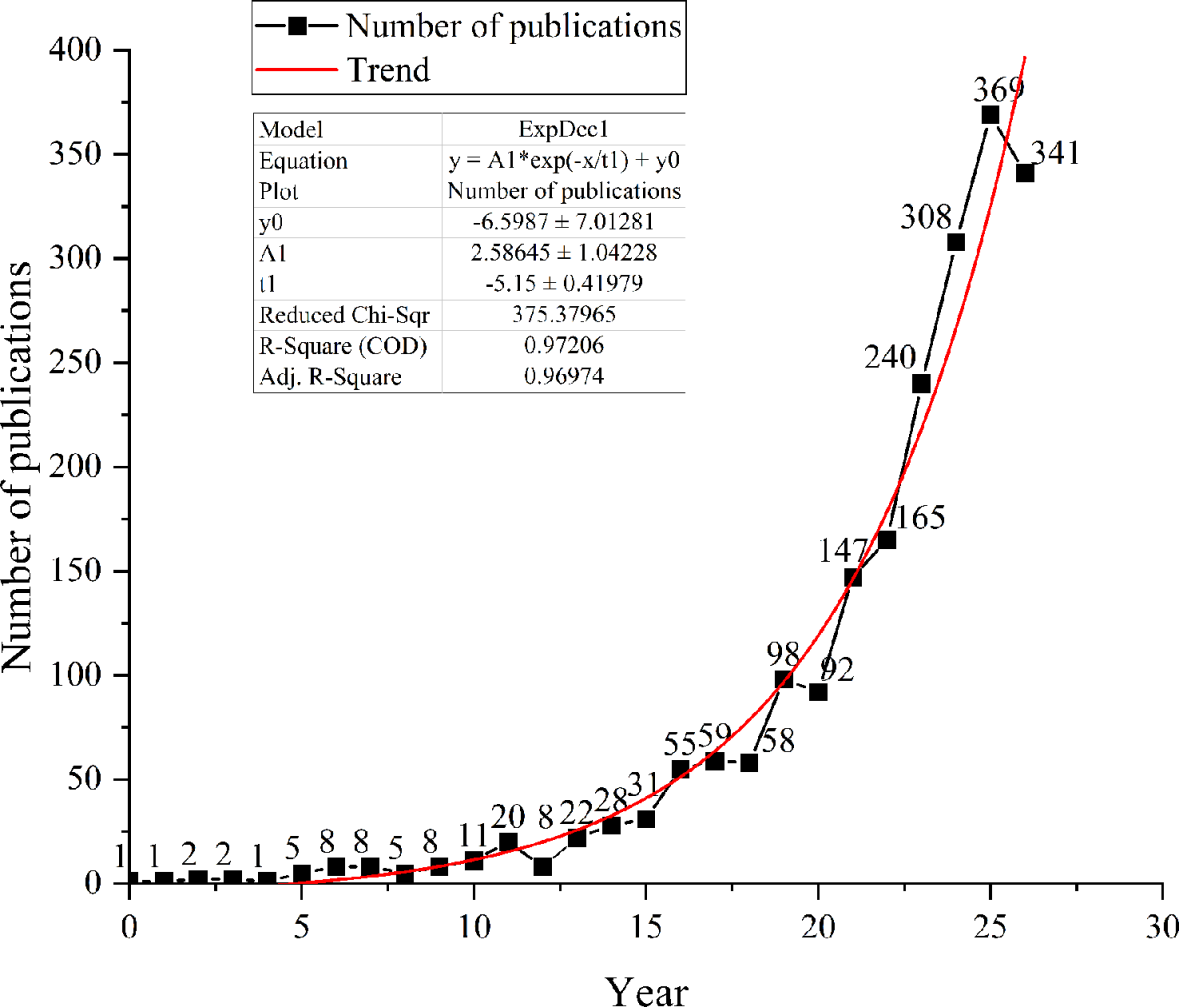
Annual publication and citation number from 1996 to 2022 at WoSCC.

The number of published papers is an essential indication of a better understanding of each stage of research development (Wang et al., 2022). The quantity of published literature that CiteSpace has counted is represented by a line graph. According to statistics of disclosure, investigations of antioxidant enzymes and substances found in microalgae began as early as 1996. The article is titled Evaluation of Active Oxygen Effect on *Chlorella Vulgaris* Photosynthesis by Hirayama, S in Free Radical Research. Examining the connection between O_2_ and an active oxygen scavenging mechanism in *Chlorella Vulgaris* is the primary objective of this study (Hirayama et al., 1996). People’s interest in this study has increased from 1996 to date, and the growth rate of cumulative publications grew steadily over time (**Figure 1**), following the equation y = 2.58645*exp(x/5.15) - 6.5987, where the year 0 is defined as 1996. The relevant research progress can be divided into three phases based on the general development pattern. The first phase, which lasted from 1996 to 2005, saw a relatively modest number of articles published annually that stabilized at around 10, with scientific activity still in its infancy. A total of 41 papers were issued during this 10-year period, representing 1.96% of the total. The number of papers published between 2006 and 2014 exhibited an upward trend, with a faster growth rate than between 1996 and 2005. A total of 292 papers were posted throughout nine years, accounting for 13.95% of the total papers, with 32 papers getting published on average per year. The number of antioxidation of high-value metabolites in microalgae research publications grew rapidly from 2015 to 2022. At this stage, known as the rapid development stage, 84.09% of all published material was accessible (Sun et al., 2019). 2015 to 2022 can be viewed as the boom stage, when an increasing number of academics began to study this topic.

### 3.2 Comparison of research outputs among various subject categories, nations and organizations

Select subject categories, countries, and institutions from CiteSpace 6.1.R3 for analysis, and set the time slice as “1996-2022”. The year of each slice is 1, and the threshold value of g index is K=25. **Table 1** collates the top ten relevant disciplines, countries and institutions in terms of the number of articles published on microalgal antioxidant research.

**Table 1:** Top 10 subject categories, countries, and institutions in terms of publications.

No.	Name	Frequency	Percentage %
Subject categories			
1	Biotechnology & Applied Microbiology	524	25.04
2	Food Science & Technology	348	16.63
3	Marine & Freshwater Biology	252	12.04
4	Environmental Science	251	11.99
5	Biochemistry & Molecular Biology	222	10.61
6	Pharmacology & Pharmacy	157	7.50
7	Chemistry, Multidisciplinary	148	7.07
8	Chemistry, Medicinal	145	6.93
9	Energy & fuels	131	6.26
10	Engineering, Chemical	127	6.07
Countries			
1	China	456	21.79
2	India	200	9.56
3	Spain	178	8.50
4	Brazil	150	7.17
5	Italy	144	6.88
6	Portugal	134	6.40
7	South Korea	113	5.40
8	USA	113	5.40
9	Egypt	105	5.02
10	France	81	3.87
Intitutions			
1	Univ Porto	50	2.38
2	Chinese Acad Sci	43	2.05
3	Ocean Univ China	36	1.71
4	CSIC	32	1.52
5	Univ Lisbon	27	1.29
6	Shenzhen Univ	26	1.24
7	Jinan Univ	25	1.19
8	Natl Cheng Kung Univ	24	1.14
9	Cairo Univ	23	1.09
10	Univ Sao Paulo	23	1.09

#### 3.2.1 Subject categories analysis

Each WoSCC article has been divided into one or more disciplinary groups. According to the number of publications in the 2093 linked articles, **Table 1** presents the top ten subject groups. The subject category with the most publications was “Biotechnology and Applied Microbiology,” which had 529. Other popular subject categories were “Food Science and Technology,” “Environmental Sciences,” “Marine and Freshwater Biology,” “Biochemistry and Molecular Biology,” etc. Among the ten topic areas, “Biochemistry & Molecular Biology” had the highest centrality (0.46), which suggested that it is more significant and influential. Overall, research on microalgal antioxidants is often an interdisciplinary endeavor combining numerous fields.

#### 3.2.2 Performance of publications by countries and regions

**Figure 2:**
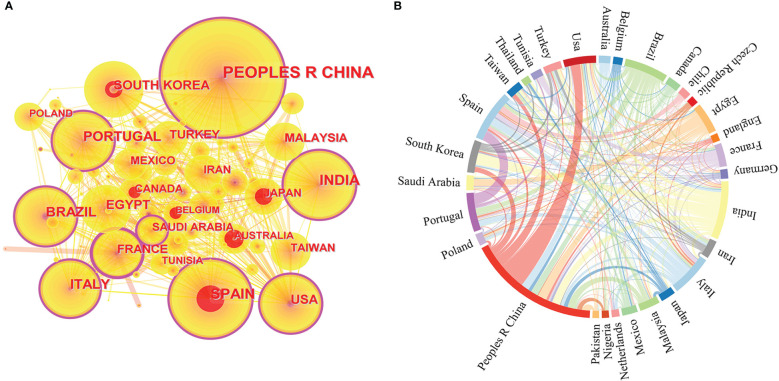
Based on the information of microalgae antioxidant research from 1996 to 2022, including **(A)** a network map of countries/regions cooperation created by CiteSpace; **(B)** a string diagram created by Gephi of the network of countries/regions cooperating.

**Figure 2A** shows that China has the largest node size indicating that China published the most publications, with 456 papers, followed by India, Spain, Brazil, Italy, and Portugal. Furthermore, the purple circle of the outer ring represents betweenness centrality, a measure of influence that reveals how closely nations, publications or journals are related to one another. A high betweenness centrality means that articles/authors from that country and region have a more significant influence on the field of study because it connects other articles/authors, and therefore more information and pathways pass through them. France (0.29) has the largest purple circle, indicating that French research in this field has been the most influential. China (0.2), Spain (0.19), Portugal (0.18), the United States (0.16), India (0.15), and Italy (0.15), Brazil (0.12), among others, also have high betweenness centrality. These countries have made a substantial contribution to the understanding of high-value metabolite antioxidation in microalgae. Finally, red citation rings indicate the research on the antioxidation of microalgae in a particular country has increased suddenly in a certain period (Chen et al., 2012).

A national cooperation network (**Figure 2B**) was developed between 1996 and 2022 for this research to better understand the number of nations and areas that have contributed to the field of study on the antioxidation of high-value metabolites in microalgae. China and the United States, Spain and Portugal, South Korea and India, Egypt and Saudi Arabia, and France and Tunisia all have significant alliances, according to an examination of the national cooperation networks.

#### 3.2.3 Most influential institutions and contributors

**Figure 3:**
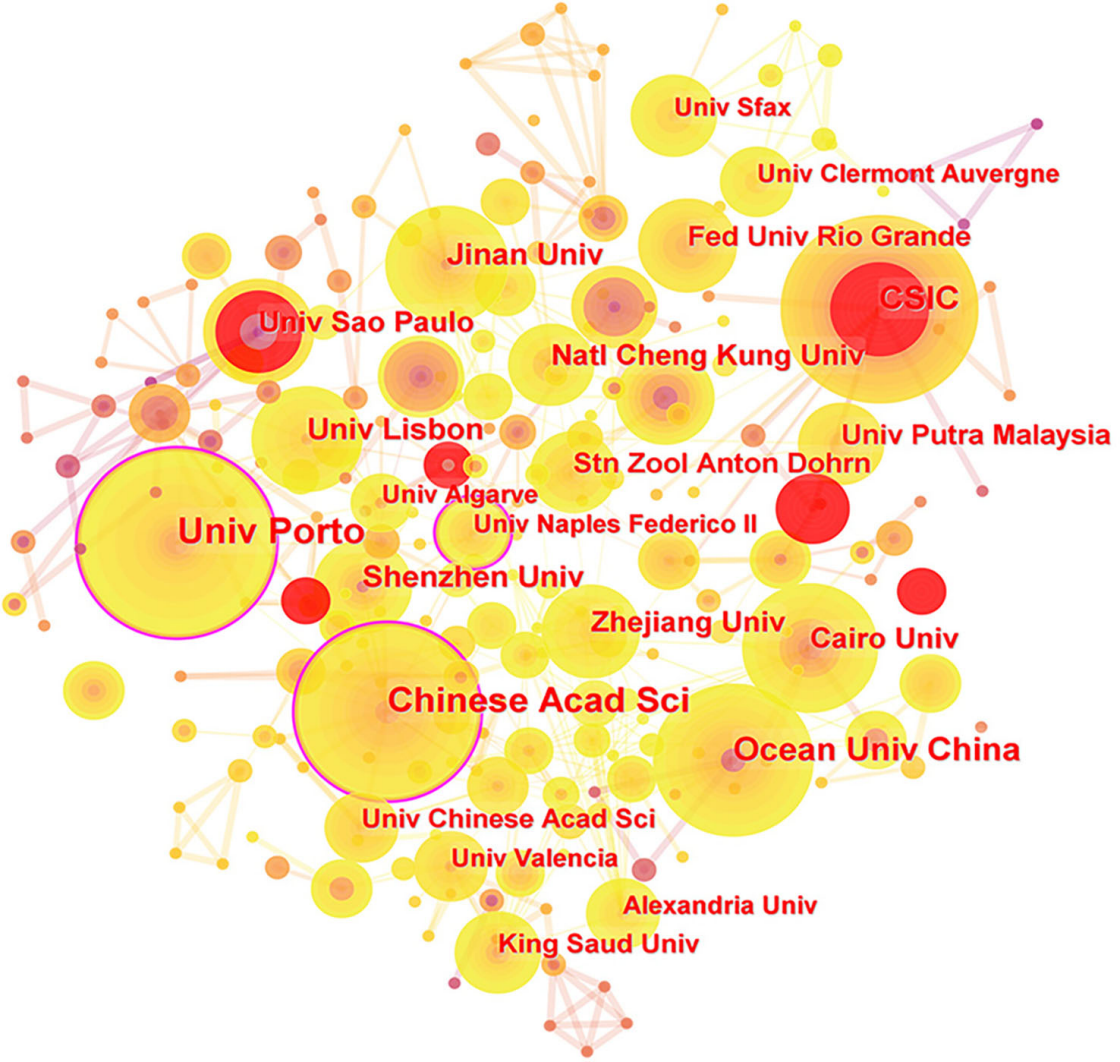
Institutions collaboration network.

CiteSpace examined 2392 institutions that had written articles about microalgal antioxidants. Among the top 10 institutions by several publications, Univ Sao Paulo’ s research on this topic was earlier, having produced literature in 1998 (Miranda et al., 1998). The University of Porto has published 48 publications, ranking first. The Chinese Academy of Sciences (43) is in second place, followed by the Ocean Univ China (32). The fact that China has the majority of the top 10 institutions demonstrates how dominant Chinese research is in this field. The University of Porto has the highest value for intermediate centrality (0.19), indicating that it has a higher impact on the development of microalgal antioxidants. The collaboration links between the institutions are depicted in **Figure 3**, with a focus on Chinese Acad Sci and Ocean Univ China, which form a research core strength and work closely with several other institutions.

**Table 2** shows the top 10 authors with the most cited research in this field from 1996 to 2022, using the total number of papers and the total number of citations worldwide as statistical indicators. Among the 8236 authors involved in microalgal antioxidant research, Ibanez, E. has the highest total number of citations (2849) and the most published papers (26 with an H-index of 21), whose main research interests are the extraction of functional components from different natural sources and the study of these components’ structure, function, and application. According to the total number of publications, other writers who have significantly advanced the study of microalgae antioxidants include Guedes, A.C., Chang, J.S., and Cifuentes, A.

**Table 2:** Top 10 authors with the most publications in microalgae antioxidant studies (based on TP).

No.	Author	TP	TC	H-index
1	Ibanez, E.	26	2849	21
2	Guedes, A.C.	24	879	11
3	Chang, J.S.	23	1674	16
4	Cifuentes, A.	20	2224	17
5	Amaro, H.M.	19	762	9
6	Costa, JAV	19	448	9
7	Malcata, F.X.	17	831	9
8	Chen, F.	15	1048	13
9	Herrero, M.	15	2120	13
10	Brunet, C.	14	485	10

TP is the total number of papers; TC is the total number of citations worldwide; the H-index indicates how many of their published papers have received at least h citations in total.

### 3.3 Knowledge structure for antioxidant research in microalgae

#### 3.3.1 Most influential institutions and contributors

The publications that receive the most citations are typically regarded as landmark pieces (Chen et al., 2012). **Table 3** shows the top 10 studies on the antioxidation of highly valuable metabolites in microalgae. Among them, the most frequently referenced literature is “The promising future of microalgae: current status, challenges, and optimization of a sustainable and renewable industry for biofuels, feed, and other products” (Khan et al., 2018), in which the advantages of microalgae for the production of biofuels and various bioactive compounds are described. Furthermore, Koyande A.K. (Koyande et al., 2019) discussed numerous microalgal proteins as well as other bio-active substances that contribute to improved health. Analysis of several other highly cited literature shows that the antioxidant capacity of bioactive substances in microalgae, especially carotenoids, phenols, and polysaccharides, and their use in industrial, food, and pharmaceutical applications is a significant study area.

**Table 3:** Documents with the highest citations in global research.

Title	Journals	Authors	Number of Citations	References
The promising future of microalgae: current status, challenges, and optimization of a sustainable and renewable industry for biofuels, feed, and other products	Microbial Cell Factories	Khan, M.I.	73	(Khan et al., 2018)
Microalgae: A potential alternative to health supplementation for humans	Food Science and Human Wellness	Koyande, A. K.	65	(Koyande et al., 2019)
Algae as nutritional and functional food sources: revisiting our understanding	Journal of Applied Phycology	Wells, M. L	55	(Wells et al., 2017)
Astaxanthin-Producing Green Microalga Haematococcus pluvialis: From Single Cellto High Value Commercia Products	Frontiers in Plant Science	Shah, M. MR	53	(Shah et al., 2016)
Carotenoids from microalgae: A review of recent developments	Biotechnology Advances	Gong, M.Y	52	(Gong and Bassi, 2016)
Microalgae biomass as an alternative ingredient in cookies: Sensory, physical and chemical properties, antioxidant activity and *in vitro* digestibility	Algal Research - Biomass Biofuels and Bioproducts	Batista, A.P.	49	(Batista et al., 2017)
Microalgae as healthy ingredients for functional food: a review	Food & Function	Matos, J	48	(Matos et al., 2017)
Microalgae metabolites: A rich source for food and medicine	Saudi Journal of Biological Sciences	Sathasivam, R.	48	(Sathasivam et al., 2019)
Trends in Microalgae Incorporation Into Innovative Food Products With Potential Health Benefits	Frontiers in Nutrition	Caporgno, M. P.	45	(Caporgno and Mathys, 2018)
Carotenoids, Phenolic Compounds and Tocopherols Contribute to the Antioxidative Properties of Some Microalgae Species Grown on Industrial Wastewater	Marine Drugs	Safafar, H.	44	(Safafar et al., 2015)

#### 3.3.2 Cluster analysis

**Figure 4:**
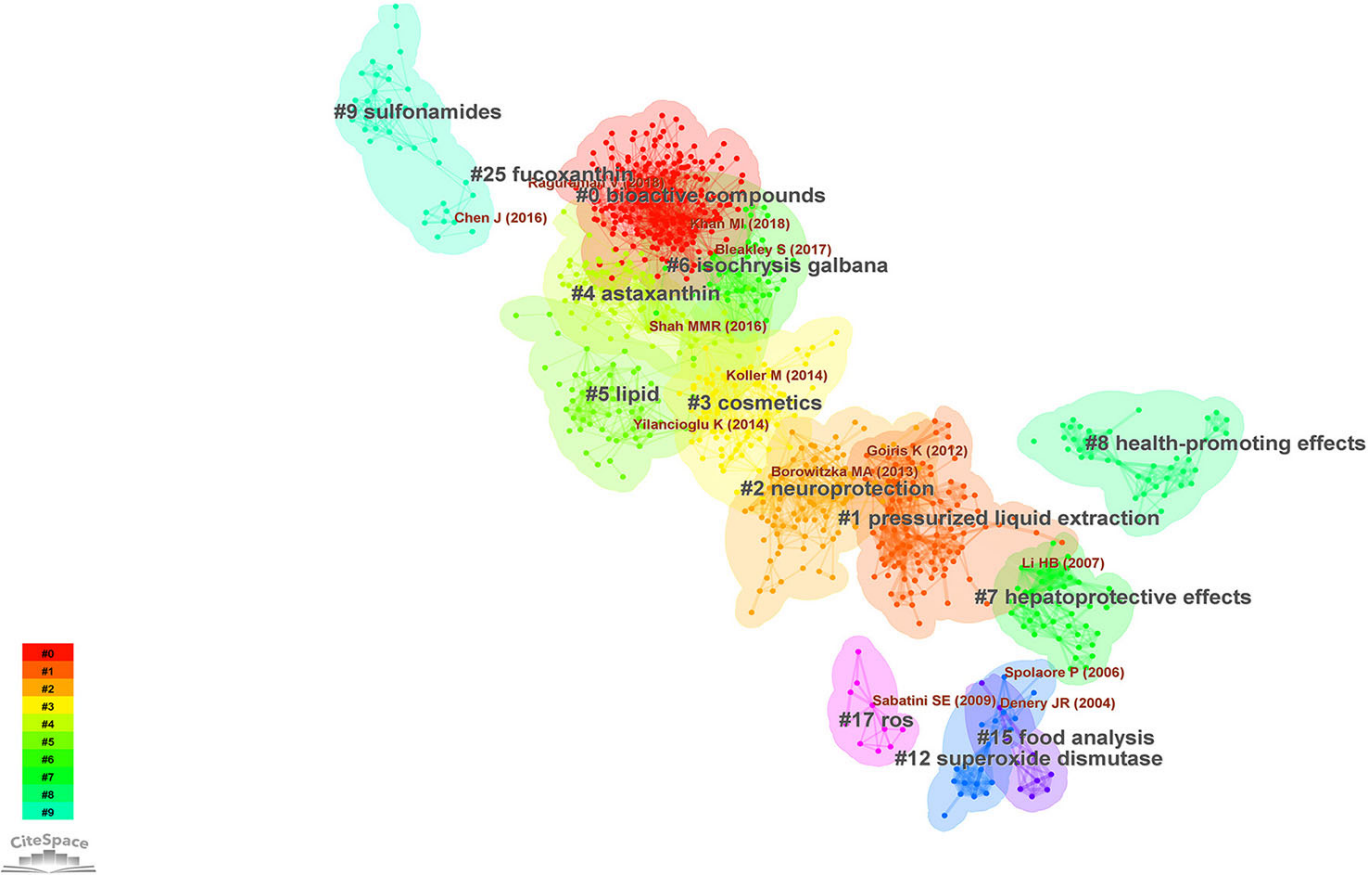
Map of the largest subnetwork of co-cited clustering of the antioxidant capacity of the bioactive substances in microalgae (Research literature from 1996 to 2022).

The document co-citation network can categorize the topics covered in this field and extract noun phrases from the titles, keywords or abstracts of articles that cite specific clusters. This study co-cited and clustered 2093 academic papers that studied microalgae antioxidants from 1996 to 2022. The clustering analysis was based on keyword clustering and used log-likelihood ratios (LLR) to provide clustering labels and visualizes the dynamics of the article clustering network using a “timeline view” and a “clustering view”. The labels for each of the 14 keyword clusters were extracted using the LLR technique (**Figure 4**). The weighted average silhouette s value was 0.8777, while the modality Q value was 0.7367. In general, the modality Q value had to be greater than 0.3 for the separation clustering structure to be significant, and the silhouette s value had to be greater than 0.5 for the clustering to be effective. The largest subnetwork had 912 nodes, representing 77% of the entire network. Specific feature information is presented in **Table 4**.

**Table 4:** Clustering information of the antioxidant capacity of the bioactive substances in microalgae.

Cluster ID	Size	Silhouette	The average research periods [Year]
0	233	0.821	2018
1	113	0.868	2008
2	103	0.872	2011
3	95	0.838	2012
4	83	0.85	2016
5	59	0.935	2015
6	51	0.885	2015
7	45	0.965	2005
8	44	0.969	2005
9	38	0.97	2017
12	21	0.991	2004
15	11	1	2002
17	10	1	2009
25	6	0.997	2018

**Figure 5:**
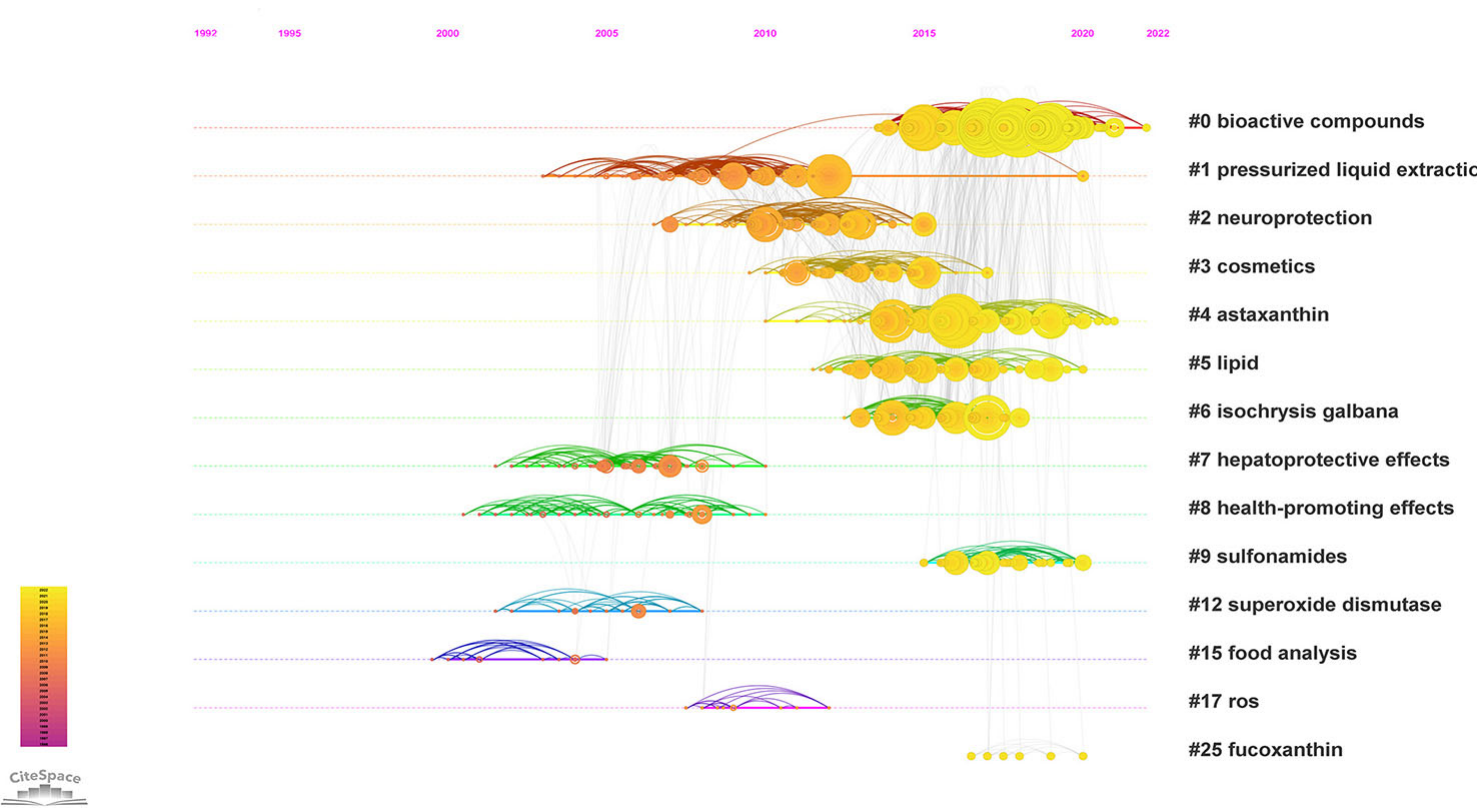
Timeline visualization with overlays of references.

**Figure 5** shows a timeline view of the co-citation network divided into clusters. The clusters are arranged in descending order according to their size when viewed from the top. Cluster *#0* studies bioactive compounds in microalgae and their relevance to health, while clusters *#4 astaxanthin*, *#5 lipid*, *#7 hepatoprotective effects*, *#8 health-promoting effects*, and *#25 fucoxanthin* are closely related to this cluster.

The research hotspots of antioxidation of high-value metabolites in microalgae mostly involve the following topics, as shown by the clustering findings and the content analysis of the literature in each cluster. (1) Reactive oxygen production, oxidative stress, and antioxidant mechanisms; (2) common assays used to assess the antioxidant activity of microalgae *in vitro*; (3) bioactive substances in different microalgae; (4) the potential use of microalgae in the food industry and in the medical, cosmetic, nutraceutical industries.

#### 3.3.3 Reactive oxygen species

**Figure 6:**
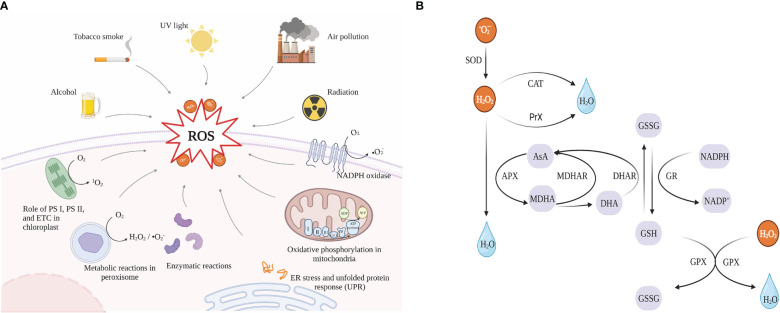
**(A)** Sources of reactive oxygen species (ROS), created with https://biorender.com/ (accessed on 24 December 2022); **(B)** combined mechanisms of enzymatic and non-enzymatic antioxidants. SOD, Superoxide dismutase; CAT, Catalase; PrX, peroxiredoxin; APX, Ascorbate peroxidase; AsA, ascorbate; MDHA, monodehydroascorbate; MDHAR, monodehydroascorbate reductase; DHA, dehydroascorbate; DHAR, dehydroascorbate reductase; GSH, reduced glutathione; GSSG, oxidized glutathione; GR, glutathione reductase; GPX, Glutathione peroxidase.

Reactive oxygen species (ROS), which include free radicals (•O_2_^-^, superoxide radicals; OH•, hydroxyl radical; HO_2_•, perhydroxy radical and RO•, alkoxy radicals) and non-free radicals (H_2_O_2_, hydrogen peroxide and ^1^O_2_, singlet oxygen) (Gill and Tuteja, 2010), are byproducts of cellular metabolism. There is convincing evidence that ROS has “two-sides” (Valko et al., 2006). At medium and low concentrations, ROS functions as a signaling factor in the physiological response of cells to hypoxia (Valko et al., 2007), and different biological systems use the mitogen-activated protein kinase (MAPK) pathway to send signals to the nucleus *via* redox reactions. However, numerous conditions can cause an organism to produce excessive amounts of ROS (**Figure 6A**), which can lead to physical damage by oxidizing proteins, damaging nucleic acids, and causing lipid peroxidation (Apel and Hirt, 2004).

Cells, however, have potent antioxidant defense mechanisms that can be separated into enzymatic systems (including SOD, APX, GPX, GST, and CAT) and non-enzymatic systems (such as ASH, GSH, alpha-tocopherol, carotenoids, and flavonoids) (Mittler et al., 2004). These systems cooperate in combating the negative consequences of ROS. The different enzymatic and non-enzymatic antioxidants that microalgae use to defend themselves against ROS damage are shown in **Figure 6B**. Microalgal bioactive components can serve as a reliable supply of antioxidants for scavenging ROS. For example, ascorbic acid (ASH, vitamin C), the most prevalent and potent water-soluble antioxidant among non-enzymatic antioxidants, is present in all plant tissues (Athar et al., 2008). Meanwhile, ASA and GSH are the two non-enzymatic antioxidants in higher plants in the most significant amounts, which play an essential role in scavenging ROS directly through the AsA-GSH cycle as electron donors (Hasanuzzaman et al., 2019). In microalgae, vitamins C and tocopherols (vitamins E) remove ROS by neutralizing free radicals with electrons, and glutathione acts as a cofactor for anti-oxidative stress detoxification enzymes and also directly scavenges hydroxyl radicals and singlet oxygen (Valko et al., 2007). In addition, according to studies, carotenoids are significant antioxidants in microalgae since they not only eliminate peroxyl and hydroxyl radicals but also act as sunscreen (Varela et al., 2015).

#### 3.3.4 Methods for the analysis of antioxidant activity

ABTS/TEAC/DPPH is the primary technique used to assess the antioxidant activity of microalgal extracts using *in vitro* chemical tests. It is important to remember that conclusions on the antioxidant activity should not be based on just one antioxidant assay but rather on a thorough assessment of the outcomes of various methods for measuring *in vitro* antioxidant activity (Alam et al., 2013). To this purpose, **Table 5** provides several frequently used assay procedures with their principles, benefits, and insufficient to giving the investigator a thorough grasp of these *in vitro* assay techniques before implementing them.

**Table 5:** Main methods for assessing antioxidant activity.

Name of the Method	Principle of the Method	Advantages	Insufficient	References
Antioxidative activity assay (ABTS)	A moderately persistent radical cation, ABTS^+^, is produced when peroxidases such as methemoglobin and H_2_O_2_ are treated with ABTS. When ABTS^+^ interacts with Ferryl myoglobin, it creates a relatively stable blue-green color that can be seen at 600 nm.	Evaluated over a wide pH range; soluble in water and organic solvents; reacts rapidly	No biological or dietary systems contain ABTS radicals, which do not represent physical components.	(Badarinath et al., 2010)
Trolox equivalent capacity assay (TEAC)	Determine the radical scavenger’s ability to reduce the blue chromophore ABTS radical cation to a colorless substance. After discoloration, an absorbance measurement is taken at 734 nm.	A proper technique to evaluate the antioxidant strength of synthetic red colorants that is easy to use, quick (6 min), and efficient.	It requires a valid calculation of the reaction’s endpoint, and the reaction rate is not represented in the TEAC values.	(Obon et al., 2005)
2,2-diphenyl-1-picrylhydrazyl assay (DPPH)	Analyze the DPPH radical’s ability to scavenge oxygen by comparing it to the light yellow compound that it transforms into when a scavenger is present. At 515 nm, the absorbance drop is gauged.	Straightforward, quick, and practical for screening a large number of samples for radical scavenging activity without regard to sample polarity.	When substances in samples absorb at the same wavelength, interference may happen, resulting in overlapped DPPH spectra.	(Alam et al., 2013)

#### 3.3.5 Microalgal intracellular compounds

The following is a discussion of some of the very valued bioactive compounds in microalgae.

The yellow and red colors that can be found in a variety of fruits and vegetables are caused by pigments that contain carotenoids (Stahl and Sies, 2003). There are two broad classifications of carotenoids: xanthophylls and carotenes. The difference between them is chemical: xanthophylls contain oxygen, while carotenes are hydrocarbons and do not contain oxygen. Carotenoids have antioxidant qualities that protect cells from reactive free radicals, prevent lipid oxidation, and possibly even promote the integrity of cell membranes (Gong and Bassi, 2016). The microalgae strains *Dunaliella salina*, *Chlorella* sp., *Haematococcus pluvialis*, and *Spirulina* sp. are ideal for producing carotenoids (Christaki et al., 2013). Naguib (Naguib, 2000) compared the antioxidant activity of various carotenoids and showed that astaxanthin (1.3) had the highest relative activity against peroxyl radicals in carotenoids, followed by 6-hydroxy-2,5,7,8-tetramethylchroman-2-carboxylic acid (1.0), α-tocopherol (0.9), lutein (0.4) and beta-carotene (0.2). This article focuses on β-carotene, astaxanthin and lutein.

The most biologically active red ketone carotenoid found in nature, astaxanthin (3,3’-dihydroxy-β-β-carotene 4,4’-dione), can be obtained from crustaceans, plants, bacteria, and fungi (Li et al., 2011). Among them, the *Haematococcus Pluvialis* is believed to be the best source of natural astaxanthin due to its astaxanthin content of up to 1-5% of cell dry weight (Wan et al., 2014). However, astaxanthin accumulation is constrained by the poor biomass productivity under normal circumstances. And under stressful conditions (high light or nutritional deficiency), fast-growing motile cells are typically used to promote astaxanthin production; however, biomass productivity is low (Hata et al., 2001). To induce astaxanthin accumulation under photoautotrophic circumstances, Zhang et al. (Zhang et al., 2016) first created the concept of heterotrophic cultures, followed by transferring the heterotrophic cells to a specific light/nutrient environment. The generation of astaxanthin was 4.2 times higher using this culture technique. Another important carotenoid pigment is β-carotene, which is mainly from *Chlorella* and *Dunaliella*. The microalga generates β-carotene in proportions equal to approximately 10–14% of its dry mass (Sathasivam et al., 2019). β-carotene content dramatically increased under photoheterotrophic circumstances: 67.5 mM acetate and 450 μM FeSO_4_ enriched growth conditions, and cellular β-carotene content rose to up to 70 pg cell^-1^ (Mojaat et al., 2008). Cations such as Fe^2+^ could increase -carotene production of β-carotene by causing oxidative stress. Due to recent investigations, zeaxanthin and the lutein are receiving more and more attention. Although it is well known that marigold resin contains antioxidants, certain microalgae have hundreds of times more lutein than marigold species (Fernandez-Sevilla et al., 2010). Numerous factors influence the lutein content of microalgae, including pH, which reduces the efficiency of CO_2_ and thus inhibits photosynthesis (Macias-Sanchez et al., 2010); high temperatures are beneficial for lutein accumulation (Fernandez-Sevilla et al., 2010), but too high temperatures are harmful. High radiation levels, the concentration of nitrogen in the medium, and the presence of oxidizing substances are partially responsible for the accumulation of lutein (Guedes et al., 2011).

Polysaccharides are heteropolymers comprising the major sugars galactose, xylose, and glucose joined together by glycosidic linkages (Chanda et al., 2019). Marine macro- and microalgae can produce a wide range of polysaccharides, either internally or externally, the majority of which are sulphated (sPS) (Raposo et al., 2015). Sulphate polysaccharides synthesized and released by marine microalgae have the ability to stop the accumulation of free radicals and reactive chemicals, according to Raposo et al. (Raposo et al., 2013). Subsequently, Tannin-spitz T et al. (Tannin-Spitz et al., 2005) corroborated this assertion and measured the ability of crude polysaccharide solutions to oxidize linoleic acid to estimate the antioxidant activity of polysaccharide solutions. The sulphated polysaccharide was shown to protect algae from ROS by scavenging free radicals and transferring them from cells to the culture medium. Likewise, the antioxidant activity is highly correlated with the content of sulfates. The heterogeneity and structural differences in the extracellular sulphate polysaccharides synthesized by different marine microalgae make research challenging. Contrary to seaweed, microalgae can be cultivated in controlled environments, improving the stability of their chemical and structural makeup (Raposo et al., 2013). Thus, microalgae polysaccharides may be essential for biobased materials in the life sciences (e.g., medical devices, pharmaceutics, food, and cosmetics).

Tocopherols, often known as vitamin E, are lipid-soluble compounds produced by photosynthesis, which act as antioxidants through single electron transfer (SET) and hydrogen atom transfer (HAT) (Herrera and Barbas, 2001). Research has shown that tocopherols can be extracted, purified, or concentrated from vegetable oils and other higher plant substances, and tocopherol-containing microalgae include *Spirulina*, *Dunaliella tertiolecta*, and *Chlorella* sp. The most active and extensively researched of the four isomers is α-tocopherol (Ogbonna, 2009). Tocopherols function as membrane stabilizers by preventing oxidative damage to vesicle-like structures (Powls and Redfearn, 1967), which play a role in the prevention of light-induced instances in the skin and eyes (Cahoon et al., 2003). The liver is one of the primary targets for ROS illness, and it has been demonstrated that phenolic substances, such as isoflavones and flavonoids, have a protective impact on the organ (Sanchez-Salgado et al., 2019). In addition, α-tocopherol may help Alzheimer’s patients feel better (Sano et al., 1997).

For tissue integrity and health benefits, polyunsaturated fatty acids (PUFAs), namely EPA and DHA, are crucial. Omega-3 and omega-6 fatty acids are necessary for the human body, but the body cannot manufacture them. The ability of numerous microalgal species (e.g. *Porphyridium cruentum*, *Arthrospira platensis*, *Odontella*, and *Isochrysis galbana*) to synthesize these critical fatty acids have been investigated (Khan et al., 2018). PUFAs are converted to triacylglycerols (TAG) or polar lipids (i.e., phospholipids, glycolipids) by the esterification of glycerol with PUFAs. These lipids offer an ideal structure to control the fluidity and function of membranes. Furthermore, the creation of additional metabolic products with high added value, such as β-carotene and astaxanthin, can be integrated with the production of lipids (Bellou et al., 2014).

#### 3.3.6 Application of bioactive substances in microalgae

Microalgae biomass should undergo an extraction procedure before use to make it a more valuable source of antioxidants (Cotas et al., 2020). Typical extraction techniques include ultrasounds methods, supercritical fluid extraction, percolation, and microwave methods. Microalgae extracts typically contain a variety of bioactive ingredients (Michalak et al., 2022). There is a trend toward using microalgae and their bioactive natural compounds in various fields, including the pharmaceutical, biomedical, cosmetic, and food industries. This research focuses on the application of the antioxidant capacity of microalgal bioactive chemicals to diverse sectors (**Table 6**).

**Table 6:** Active antioxidant functional ingredients in common microalgae.

Functional Ingredient	Microalga Source	Applications Related to Oxidation Resistance	References
phycobiliproteins	*Spirulina platensis*	prevent some diseases and damage in cells/tissues	(Madyastha, 2000; Romay et al., 2003)
β-carotene	*Dunaliella salina;* *Haematococcus*	food additives; multivitamin preparations	(Guedes et al., 2011)
Lutein	*Chlorella vulgaris;* *Muriellopsis* sp.*;* *Scenedesmus almeriensis;*		
Astaxanthin	*Haematococcus pluvialis;* *Chlorella zofingiensis; Chlorococcum* sp.	Protect against UV-induced photooxidation; protect against gastric lesions (ulcers)	(Shah et al., 2016)
oleic acid	*Spirulina platensis;* *Dunaliella salina*	Prevent or treat cardiovascular Disorders.	(Herrero et al., 2006)
tocopherols	*Spirulina platensis;* *Dunaliella tertiolecta;* *Synechocystis* sp.*;* *Nannochloropsis oculata*	Protect membrane lipids from oxidative damage.	(Vismara et al., 2003; Ogbonna, 2009)
polysaccharide	*Porphyridium* sp.	protect mouse cells and tissues against oxidative damage;	(Sun et al., 2009)
phenolic compounds	*Spirulina platensis*	Sunscreen	(Klejdus et al., 2009)

The benefits of microalgae as a new functional and healthy element are becoming more well known. Patients at risk of macular degeneration can avoid the danger by giving them lutein in the form of a nutritional supplement (Fernandez-Sevilla et al., 2010). As most of the sPS in microalgae have strong antioxidant properties, they may protect human health from ROS that cause cancer, diabetes, some inflammatory and neurodegenerative diseases, and other ageing-related diseases such as Alzheimer’ s disease and cardiovascular disease (Raposo et al., 2015). Long-chain poly unsaturated fatty acids (LC-PUFA), such as docosahexanoic acid (C22:6) and eicosapentaenoic acid (C20:5), are good for cardiovascular health (Harris et al., 2008). Studies conducted *in vivo* and *in vitro* have demonstrated that astaxanthin works well to stop the oxidation of low-density lipoprotein (LDL), which is utilized for safeguarding the body against various ailments (Riccioni et al., 2011). Several studies have recently demonstrated that the addition of astaxanthin to the human diet can reduce oxidative stress and further improve the immune system in patients with cardiovascular disease (Park et al., 2010). Consumption of astaxanthin erythrocytes may prevent oxidative damage in heavy smokers by inhibiting lipid peroxidation (Kim et al., 2011).

In 1952, the Algae Mass-Culture Symposium proposed using microalgae for biochemical and dietary purposes (Caporgno and Mathys, 2018). Even though some beneficial microalgae cannot currently be used directly in food, several bioactive chemicals derived from these sources have been authorized for use in human or animal diets. The antioxidant functional components of the microalgal active ingredients have also received attention due to their potential for use as preservatives and antioxidants in the food industry to lengthen the shelf life of foods (Lourenco et al., 2019) as well as their ability to be added to foods to improve human and animal nutrition (Carocho et al., 2018). For instance, when farmed marine animals eat feed containing astaxanthin, their health, and nutritional quality also advance in addition to their appearance. In addition, roughly ten different microalgae or microalgae extracts can be used as food or components in Europe (Coulombier et al., 2021). Protein is currently the primary source of protein for food and feed, and algae may account for up to 18% of protein sources by mid-century. It was suggested several decades ago to add *Dunaliella* as a protein supplement to white wheat bread (Finney et al., 1984). Furthermore, a recent development in the food sector is the blend of inorganic nanoparticles (NP) and natural antioxidant chemicals. Due to the more robust antibacterial, antioxidant, UV barrier, oxygen removal, and low environmental impact properties of these compounds, their inclusion in biological and biodegradable matrices such as polysaccharides and proteins emphasizes the potential application of active food packaging (Vieira et al., 2022). However, due to a lack of technology and economies of scale in their production and processing, employing microalgae and their bioactive compounds as food alternatives presents hurdles (Caporgno and Mathys, 2018).

Algae are naturally exposed to oxidative stress and have developed several effective protective systems against ROS and free radicals, producing compounds that can be used in cosmetics to combat the harmful effects of UV radiation (Ariede et al., 2017). *Chlorella vulgaris* and *Spirulina obtusifolia* contain chlorophyll and carotenoids, due to their antioxidant capabilities, these compounds may help prevent oil oxidation in formulations, particularly in emulsions with many oily phases (Gouveia et al., 2008). Additionally, red microalgae extracts are used in skin care, sun protection, hair care, emollients, regeneration care products, and anti-aging lotions (Borowitzka, 2013). Furthermore, microalgae and their bioactive substances have been reported to be added mainly to cosmetic formulations as thickeners and water-binding agents (El Gamal, 2010) in addition to being an antioxidant, such as specific components of the microalgae extract react with various proteins on the skin to form a protective gel on the skin’s surface, preventing moisture loss (Yarkent et al., 2020).

It should be noted that thorough scientific trials are required for natural antioxidants before microalgae and its bioactive components can be used in food, medicine, or cosmetics. To effectively utilize natural antioxidants, clarifying their structural properties and removing any undesirable components is necessary (Rani et al., 2021).

### 3.4 Analysis of keyword co-occurrence and burst keywords

As descriptive terms, keywords can be employed to analyze the historical development of a subject area (Yu et al., 2017). In this section, we examine the content by examining the keyword distribution. It will display the keywords co-occurrence network map, the keywords density visualization map, and the keywords with the strongest citation burst view.

#### 3.4.1 Keyword analysis

**Figure 7:**
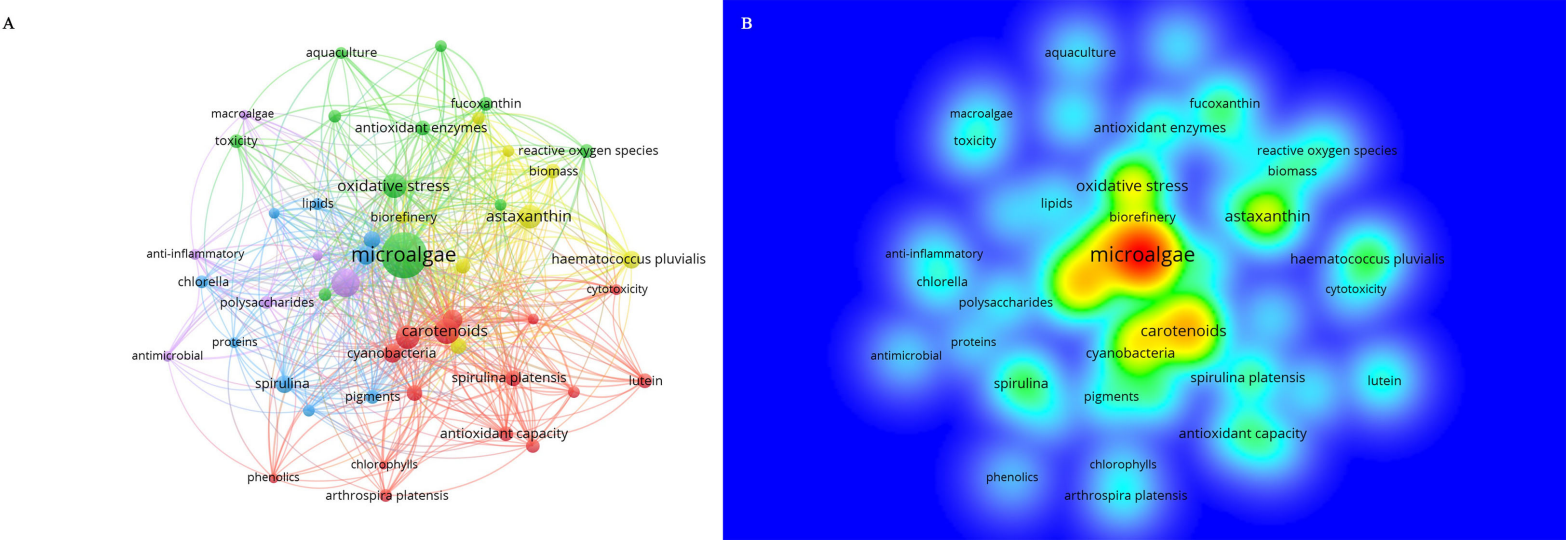
**(A)** Top 50 keywords most prominently displayed in VOSviewer; **(B)** visualization of keyword density in microalgal antioxidation study.

The program VOSviewer built a network of co-occurring author keywords on antioxidants in microalgal bioactives. A total of 4750 authors’ keywords were used, and the top 50 most frequently occurring were selected to create **Figure 7**. The size of the nodes and words in **Figure 7A** corresponds to the nodes’ weights. The distance between two nodes indicates how closely they are connected. Generally, a lesser distance indicates a closer bond. A line between two keywords demonstrates that they have occurred together, and the more co-occurrences there are, the thicker the line gets (Gu et al., 2017). Keywords that appear more frequently are “microalgae,” “carotenoids,” “astaxanthin,” “bioactive compound,” and “oxidative stress”. Additionally, a density map of the top 50 keywords used by the writers was made using VOSviewer, with redder and brighter colors denoting greater amounts of research. **Figure 7B** shows that in addition to the more well-known keywords, “polysaccharides,” “lutein” and “antioxidant enzymes” also show up in the plots and might be of particular interest for further study.

#### 3.4.2 Burst detection analysis

**Figure 8:**
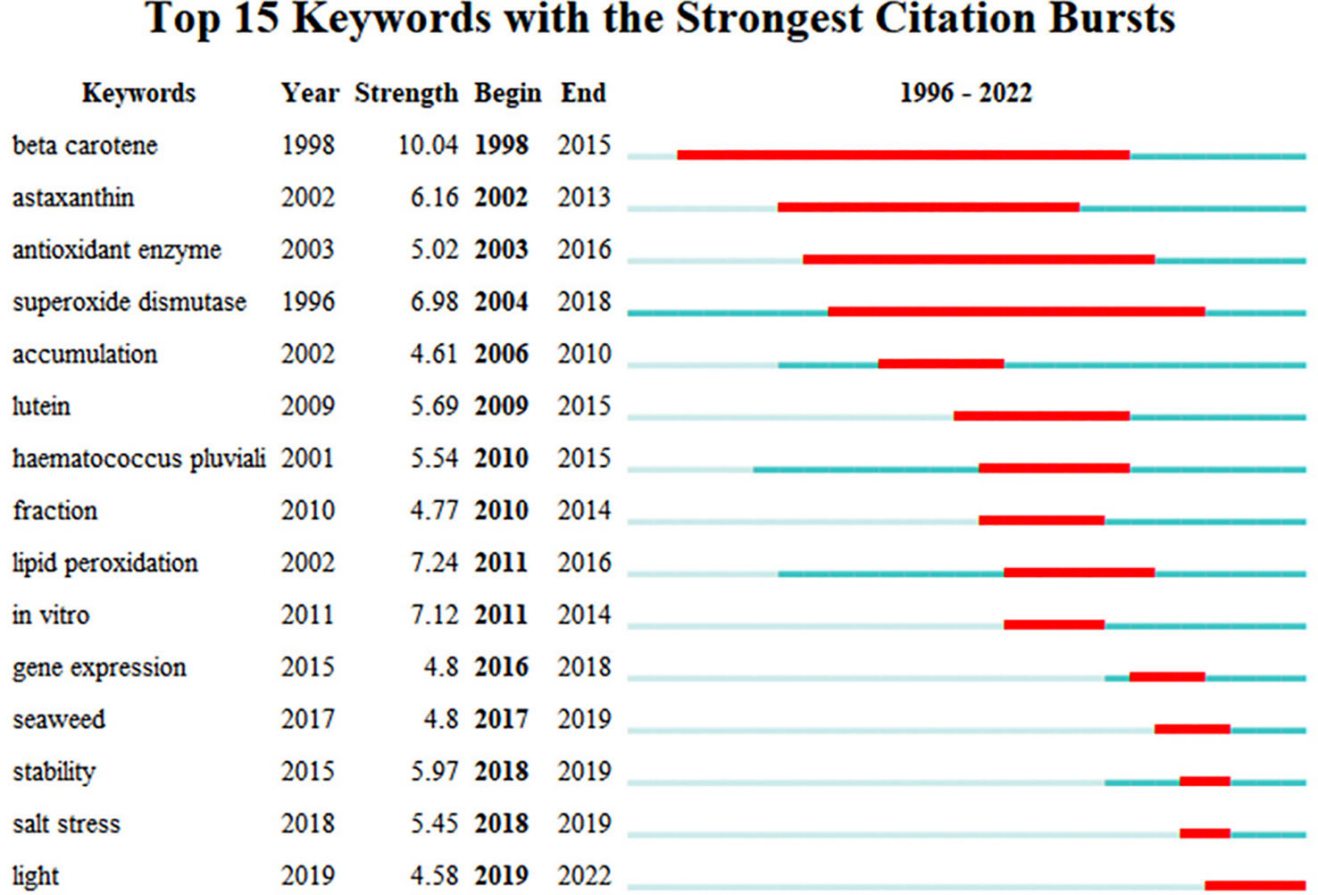
The top 15 keywords with the strongest citation burst.

From 1996 to 2022, the keywords “microalgae metabolites antioxidation research” appeared to have a high citation burst (**Figure 8**). The blue lines represent this period. The starting and ending years of the burst duration are indicated by a red line segment that represents the time period during which a subject category was discovered to have a burst (Chen et al., 2014). The citation explosion started in 1998 for beta-carotene, with the highest explosion intensity (10.04) and the longest explosion time, cited for 17 years from 1998 to 2015. Astaxanthin, the term that came after the epidemic in 2002, was mentioned to have a 15-year duration and a 6.16 burst strength. Superoxide dismutase and antioxidant enzyme citation bursts persisted for more than ten years. It is worth noting that gene expression, seaweed, stability, salt stress, and light have emerged as a new field of study in recent years, while accumulation, lutein, *Haematococcus pluvialis*, fraction, lipid peroxidation, *in vitro* have been a research focus from 2009 to 2016.

### 3.5 Emerging trends and outlook

#### 3.5.1 Analysis of research trend

Fucoxanthin, polysaccharides, salt stress, and light exposure are predicted to be the topics of future research based on keyword analysis.

Fucoxanthin, one of the carotenoids, as a natural pigment that belongs to the category of xanthophylls, is present exclusively in algae. Due to its numerous biological and health-promoting qualities, including its anti-diabetic, anti-obesity, anti-cancer, and antioxidant characteristics, fucoxanthin has received much attention lately (Muthuirulappan and Francis, 2013). The amount of fucoxanthin in microalgae has been shown to grow at light densities between 10 and 100 µmol/m^2^/s; however, the effects of varying light, nutrition, salinity, temperature, and carbon dioxide on fucoxanthin levels vary (Khaw et al., 2022). Therefore, investigations on temperature and salinity are required to achieve the ideal balance between fucoxanthin and biomass production while swiftly putting fucoxanthin into commercial production. For example, studies have been done to increase the stability and bioavailability of extracts such as astaxanthin by using nanoencapsulation methods. In the future, combining fucoxanthin with nanotechnology is possible, but only if it’s essential to prove that such effects are being exerted before usage and not toxic ones that are dangerous to people.

“Polysaccharide” also appears and shows some intensity in the keyword density graph. Microalgae polysaccharides can prolong the shelf life of vegetables and fruits and are mostly employed in food science and technology for health products, food safety, food coating preservation, food additives, and other purposes. Combining inorganic nanoparticles (NPs) with natural antioxidant compounds is a trend in the food industry that emphasizes the potential uses for active food packaging.

#### 3.5.2 Defects and difficulties

The objective data analysis was the foundation for the conclusions, but certain restrictions exist. First, we used the relatively homogeneous original English language literature from the WoSCC database. In addition, the use of other databases and publications in other languages at the same time may improve the rigorism. Second, the primary focus of this article is on the antioxidant properties of bioactive substances, but additional research on the functions and mechanisms of some essential antioxidant enzymes is warranted; one such enzyme is SOD, which serves as the body’ s first line of defense against ROS mediation and is crucial in treating diseases linked to oxidative stress. Third, carotenoids play a significant role in microalgae, which has been revealed by the bibliometric examination of the antioxidant capacity of bioactive compounds in microalgae. The varieties of microalgae species that produce lutein and carotene, as well as their use, should be examined more thoroughly in subsequent research. Finally, a change in scientific interest in antioxidant chemicals has been noticed, with a steady shift from the research of antioxidant vitamins and minerals to the study of antioxidant phytochemicals. From choosing the most suited microalgal strain to growth techniques and downstream processes, several sequential actions must be taken to produce bioproducts from microalgal biomass (e.g., harvesting, pretreatment, extraction, and purification). Although these treatments can improve the number of bioactive compounds in microalgae, they also impede the growth of microalgal biomass, somewhat restricting the commercial and industrial use of microalgae. The future trend will be to identify appropriate culture settings to increase extraction rates and, at the same time, to identify hydroxyl groups or proteins that can bind to the bioactive components of microalgae and increase the stability of those components through combinations.

## 4 Conclusion

This paper uses a bibliometric approach to evaluate trends and hotspots related to bioactive substances and antioxidant properties of microalgae. Between 1996 and 2022, 2093 original articles and reviews were retrieved. The field was found to have published few papers until 2006, gradually increasing through 2014 and then development in a period of rapid growth, with the most productive period currently occurring in 2021. Indicating that this field is an area of ongoing research interest, we speculate that antioxidant research on the bioactivity of microalgae will remain a hot research topic in the future.

Regarding national research, the number of global publications is unevenly distributed between countries, with only nine countries publishing more than 100 articles over 27 years. It is interesting to note that France, in tenth place in terms of several publications, surpassed China in terms of the number of cited times, ranking first. This might result from early research and tight relationships between France and other nations. As a result, international interaction and collaboration should be enhanced, and transnational and cross-team collaboration is key to accelerating research development. Regarding the results of the institutional analysis, the University of Porto and the Chinese Academy of Sciences show a substantial contribution in this field, with a high-citation burst from CSIC and the University of So Paulo. And finally, according to the keyword analysis, the most explored topic is the characterization of bioactive substances, and extraction and application are the focus of future research.

Overall, by systematically summarizing the literature in this field, this study demonstrates the dynamic development process and structural relationships of knowledge in related disciplines in data visualization and explores some hotspot areas. It is recommended to maintain cooperative exchanges between countries, institutions in the field of bioactive antioxidant function of microalgae substances, to focus on scientific hotspots and to promote the academic development of the area jointly.

## Author contributions

NY: Methodology, Software, Validation, Investigation, Writing - Original Draft. QZ: Methodology, Software, Validation, Investigation. CJ: Formal analysis, Investigation, Resources. SW: Investigation, Resources. RC&LY: Data Curation, Visualization. BL: Writing - Review & Editing. XL: Supervision, Project administration. RZ: Supervision, Project administration. ZZ: Conceptualization, Resources, Writing - Review & Editing, Funding acquisition.All authors contributed to the article and approved the submitted version.
